# A Decision on Medical Practice Acts

**Published:** 1889-02

**Authors:** 


### A DECISION ON MEDICAL PRACTICE ACTS.

The Supreme Court of the United States has recently delivered, through Mr. Justice Field, an important decision in regard to the validity of statutes regulating the practice of medicine. The decision was rendered in a case appealed from the Supreme Court of West Virginia, an irregular practitioner having been prosecuted in that State without a diploma or a certificate from the Board of Examiners, at the same time not being exempt under the ten years' clause of the West Virginia Act.

The case involved the validity of the Act, and the United States Supreme Court decided as follows: "The power of the State to provide for the general welfare of its people authorizes it to prescribe all such regulations as may be necessary to secure the people against the consequences of ignorance and incapacity as well as of deception and fraud. One means to secure this end is the method adopted by the State of West Virginia. If the means adopted are appropriate to the calling or profession, and obtainable by reasonable study or application, no objection to their validity can be raised."

